# A little less aggregation a little more replication: Viral manipulation of stress granules

**DOI:** 10.1002/wrna.1741

**Published:** 2022-06-16

**Authors:** Matthew J. Brownsword, Nicolas Locker

**Affiliations:** ^1^ Faculty of Health and Medical Sciences, School of Biosciences and Medicine University of Surrey Guildford Surrey UK

**Keywords:** G3BP1, phase separation, stress granules, stress response, virus

## Abstract

Recent exciting studies have uncovered how membrane‐less organelles, also known as biocondensates, are providing cells with rapid response pathways, allowing them to re‐organize their cellular contents and adapt to stressful conditions. Their assembly is driven by the phase separation of their RNAs and intrinsically disordered protein components into condensed foci. Among these, stress granules (SGs) are dynamic cytoplasmic biocondensates that form in response to many stresses, including activation of the integrated stress response or viral infections. SGs sit at the crossroads between antiviral signaling and translation because they concentrate signaling proteins and components of the innate immune response, in addition to translation machinery and stalled mRNAs. Consequently, they have been proposed to contribute to antiviral activities, and therefore are targeted by viral countermeasures. Equally, SGs components can be commandeered by viruses for their own efficient replication. Phase separation processes are an important component of the viral life cycle, for example, driving the assembly of replication factories or inclusion bodies. Therefore, in this review, we will outline the recent understanding of this complex interplay and tug of war between viruses, SGs, and their components.

This article is categorized under:

RNA in Disease and Development > RNA in Disease
Translation > Regulation
RNA Interactions with Proteins and Other Molecules > RNA‐Protein Complexes

## Graphical abstract

Viruses depend entirely on host machinery and therefore aim to conquer the host and defeat its defenses to co‐opt its resources for its own replication. Improved understanding of how viruses counteract host immune responses such as stress granule assembly, detailed here, will inform future antiviral therapeutic strategies.

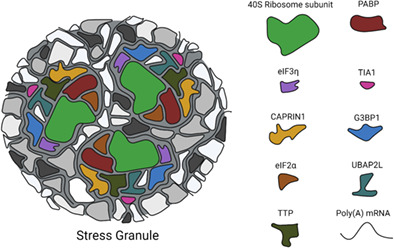

## 1 INTRODUCTION

Controlling the localization and function of macromolecules is central to cellular homeostasis. This can be achieved by surrounding them with lipid membranes in organelles such as the nucleus, lysosomes, or mitochondria. However, in recent years, the assembly of membraneless compartments, known as biocondensates or membraneless organelles, has been increasingly recognized as an alternative way to organize cellular components. These biocondensates are maintained through a combination of protein–protein, protein–RNA, and RNA–RNA interactions which result in increased local concentrations of client RNAs and proteins (D. Tauber et al., 2020). Because of this remarkable property, biocondensates provide an ideal scaffold for the regulation of fundamental processes, such as mRNA metabolism or intracellular signaling, and help cells to adapt in response to various physiological and pathological triggers (Yoo et al., 2019). Stress granules (SGs) are among the most characterized cytoplasmic biocondensates (Corbet & Parker, 2019; Hofmann et al., 2020). They capture mRNAs and proteins during different stresses including oxidative stress, heat shock, viral infection, proteasomal inhibition, ER stress, UV irradiation, among others (Hofmann et al., 2020). At first, the stress induces the global shut‐off of translation, resulting in the dissociation of mRNAs from polysomes and their accumulation in RNP complexes (Hofmann et al., 2020). The increased concentration of cytoplasmic RNPs and their binding by aggregation‐prone RNA‐binding proteins (RBPs), such as Ras‐GTPase activating SH3 domain‐binding protein 1 (G3BP1) and T‐cell internal antigen‐1 (TIA‐1), triggers the recruitment of multiple proteins characterized by the presence of low‐sequence complexity, intrinsically disordered regions (IDRs) in their structures. These IDRs mediate clustering/fusion events driven by multivalent interactions between their protein and RNA components, with G3BP1 acting as a key node for promoting RNA–protein, protein–protein, and RNA–RNA interactions, ultimately resulting in liquid–liquid phase separation (LLPS) and SG formation (Corbet & Parker, 2019; Wang et al., 2018). SGs are highly dynamic, rapidly assembling and dissolving upon stress resolution to release the stored mRNAs for translation re‐entry (Matheny et al., 2021; Namkoong et al., 2018). By sequestering specific proteins, it has been proposed that SGs regulate the composition and concentration of cytoplasmic proteins, in turn affecting the course of biochemical reactions and signaling cascades (C. L. Riggs et al., 2020). Moreover, mutations impacting SG clearance or dysregulating LLPS, can lead to persistent or aberrant SGs, which are increasingly associated with neuropathology, in particular, amyotrophic lateral sclerosis (ALS) and related diseases (Wolozin & Ivanov, 2019). Many SG proteins are also aberrantly expressed in tumors and SGs are exploited by cancer cells to adapt to the adverse conditions of the tumor microenvironment (P. Anderson et al., 2015).

SGs sits at the crossroads between intracellular signaling, antiviral responses, and translational control. Indeed, recent work has proposed that SGs exert antiviral activities by concentrating key signaling and cytoplasmic sensors or effectors of innate immunity (Eiermann et al., 2020; Mateju & Chao, 2021). The induction of type I interferons (IFNs) represents a first line of defense against pathogens, and multiple IFN signaling molecules can be recruited to SGs, with their SG localization proposed to regulate their activity. Furthermore, SGs or specific antiviral SGs (avSG) have been proposed to contribute to antiviral signaling to amplify the activation of innate immunity sensors (Eiermann et al., 2020; Mateju & Chao, 2021). Despite many studies showing increased antiviral responses when SG assembly is allowed, many such studies have used viral mutants (NS1 mutant of influenza) to investigate the antiviral nature of SGs as most viruses inhibit SGs or avoid sequestration by its factors. Therefore, viral models in which native avSGs can be studied would be welcome to elucidate this area. Therefore, because of this proposed role in antiviral signaling and impact on cellular protein synthesis which they rely on, many viruses have evolved strategies to antagonize or exploit SGs, which will be extensively discussed in this review, for example, by cleaving or repurposing SG‐nucleating proteins during infection or impairing the SG sensing pathway.

## 2 TRANSLATIONAL CONTROL DURING VIRAL INFECTION

Viruses are obligate intracellular parasites and therefore are strictly dependent on the host translation system to produce viral proteins and replicate. Because of this dependency, viruses have evolved specialized mechanisms to hijack the host machinery and ensure efficient gene expression. Moreover, the tight competition between viral and cellular translation is illustrated by the opposing need of synthesizing viral proteins to ensure efficient replication and cellular proteins that mediate the antiviral responses. Therefore, both viruses and infected hosts mediate an arsenal of regulatory mechanisms of translational control to disable the production of antiviral proteins and favor viral translation (virus‐led) or shut‐off global translation to prevent the production of viral proteins while favoring the production of essential genes (host‐led). These mechanisms have all been reviewed extensively in recent years and will only be introduced here in the context of the factors involved in the downstream assembly of SG (reviewed by Eiermann et al. (2020), McCormick and Khaperskyy (2017), and Q. Zhang, Sharma, et al. (2019)).

### 2.1 Overview of the initiation of translation

Translation can be divided into three stages, initiation, elongation, and termination (Gebauer & Hentze, 2004). Among these, initiation is a complex event requiring the coordinated action of over 25 proteins. This complexity makes initiation the most highly regulated phase of translation and its strict regulation is essential as this process accounts for a large proportion of the cells energy budget (Hershey et al., 2012).

Translation initiation begins with the recruitment of the 43S complex (made up of a 40S ribosomal subunit bound to eukaryotic initiation factor 2 [eIF2]‐GTP/Met‐tRNAi, eIF1A, eIF1, and eIF3) by eIF4F, which recognizes the 5′‐cap (Pacheco & Martinez‐Salas, 2010). The eIF4F complex is composed of eIF4E (the cap‐binding protein); eIF4A (the RNA helicase required during ribosome scanning to unwind the RNA), and the scaffolding protein eIF4G (strongly associates with eIF4E and interacts with eIF4A and eIF3; Hinnebusch, 2014; Pacheco & Martinez‐Salas, 2010). Poly(A)‐binding protein (PABP) is recruited to the polyadenylated tail of the mRNA and interacts with eIF4F. The 43S pre‐initiation complex scans the mRNA 5′ to 3′, following the helicase activity of eIF4A and its cofactor eIF4B, until the AUG initiation start codon is identified. This process is aided by additional factors, eIF1, eIF1A, eIF2, and eIF5. These factors then form the 48S complex upon recognition of the AUG start codon (Pacheco & Martinez‐Salas, 2010). The ternary complex composed of eIF2, GTP, and the initiator methionyl tRNA (Met‐tRNA) delivers the latter to the ribosome following a GTP hydrolysis step mediated by the GTPase activating protein eIF5 (Sidrauski et al., 2015). Finally, eIF5B mediates the formation of the protein synthesis competent, 80S complex by driving the joining of the 60S and 40S subunits through another round of GTP hydrolysis (Pacheco & Martinez‐Salas, 2010). When capped and polyadenylated, viral mRNAs can be translated following a similar process. However, the translation of some viral mRNAs is achieved through cap‐independent mechanisms that mostly rely upon cis‐acting secondary or tertiary RNA structures. These include internal ribosome sites (IRES; reviewed here; Lee et al., 2017), tRNA‐like structures, 3′‐cap‐independent translation elements, programmed ribosomal frameshifting signals, and other signals mediating stop‐codon readthrough or termination‐dependent reinitiation (Jaafar & Kieft, 2019). Poliovirus and encephalomyocarditis virus were the first examples of IRES elements in picornaviruses (Jang et al., 1988; Pelletier & Sonenberg, 1988). Besides RNA viruses, a wide range of IRES elements have also been found in other viruses including retroviruses and DNA viruses (Lee et al., 2017).

### 2.2 Inhibition of translation initiation during viral infection

Viruses are obligate intracellular parasites which are utterly dependent on host translation machinery, therefore must ensure control of the translation machinery to replicate efficiently. Innate host defenses counteract this by selectively or globally impairing the translation machinery in response to the detection of conserved viral nucleic acid features, such as uncapped single‐stranded RNA, dsRNA, or cytoplasmic DNA [reviewed in Eiermann et al. (2020) and Stern‐Ginossar et al. (2019)]. Protein synthesis is an energy‐intensive process for the cell, which must be tightly regulated to ensure efficient gene expression and preserve cellular resources. Specific mechanisms enable cells to rapidly reprogram protein synthesis in responses to stressful conditions, including viral infection. Most of these mechanisms specifically regulate translation initiation by either preventing the recycling of the eIF2‐GDP (guanosine diphosphate) or by disrupting the eIF4F complex (Jackson et al., 2010).

#### 2.2.1 eIF2α phosphorylation

**FIGURE 1:**
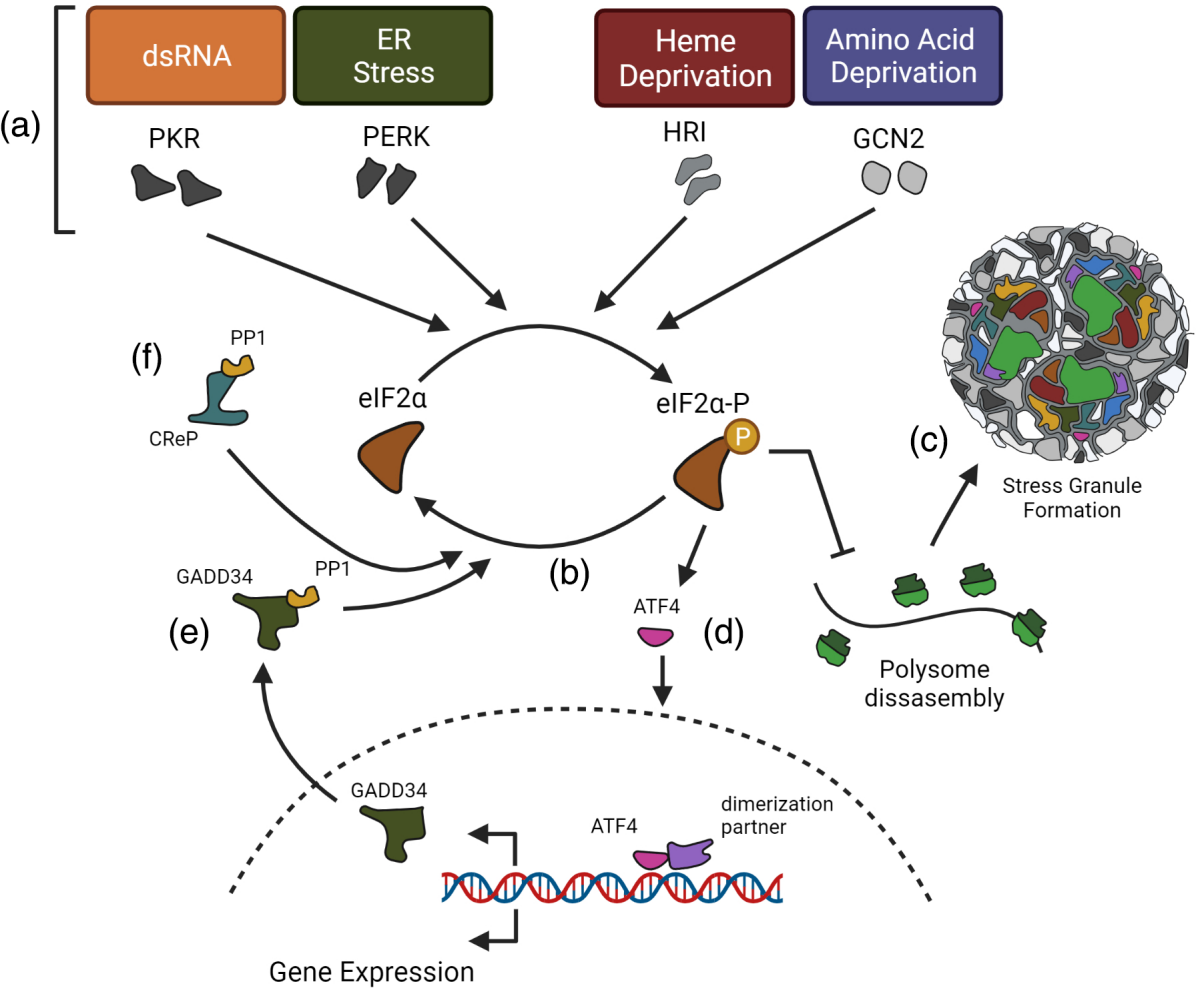
Schematic of the integrated stress response and stress granule formation. (a**)** eIF2α is phosphorylated by one of four eIF2α kinases, which are each by different types of stress. The heme‐regulated inhibitor (HRI) senses oxidative stress, osmotic stress, heat shock, and heme depletion. PERK, a transducer of the unfolded protein response (UPR), is activated by the accumulation of misfolded proteins in the ER. PKR is activated by cellular and viral dsRNAs. Finally, the general control nonderepressible 2 (GCN2) is mainly activated by limited amino acid availability and UV stress. (b) Under stress conditions, the phosphorylation of eIF2α leads to an inhibition of global translation with the exception of a subset of genes necessary for cell survival and response to stress. (c) Stress granules assemble following the release of free RNA from the polysomes. (d) Translation of genes such as ATF4 continues despite translation inhibition and functions to activate the expression of other integrated stress response genes such as GADD34 (e) which associates with PP1 to dephosphorylate eIF2α. (f) Constitutive repressor of eIF2α phosphorylation (CreP) is constitutively expressed and maintains low levels of phosphorylated eIF2α. Figure made using Biorender.

Phosphorylation of the alpha subunit of initiation factor, eIF2 (eIF2α) at residue Ser‐51, plays a key role in translational control during stress. This is achieved by one of four eIF2α serine/threonine kinases (Figure 1a), which include PKR activated by double‐stranded RNA (Proud, 1995), PERK, activated by ER stress such as unfolded protein accumulation (Harding et al., 1999), heme‐regulated inhibitor (HRI) activated by heat shock or oxidative stress (Lu et al., 2001; McEwen et al., 2005) and general control nonderepressible protein 2 (GCN2) activated by amino acid or serum deprivation and UV radiation (Berlanga et al., 1999; Deng et al., 2002; Harding et al., 2000). Additionally, HRI is thought to be responsible for the activation of the cytosolic UPR (cUPR) which manages the folding and scaffolding of innate immune sensors into signalosomes, becoming activated upon assembly of toxic superstructures. These can develop when the assembly of pattern recognition molecules into larges complexes is unchecked. The cUPR is thought to be important in the pro‐aggregative diseases such as those seen in neurodegeneration (Abdel‐Nour et al., 2019; Mukherjee et al., 2021). EIF2, part of the eIF2‐GTP‐Met‐tRNA^Met^ ternary complex, mediates binding of the Met‐tRNA with the 40S subunit in a GTP‐dependent process (Jackson et al., 2010). Under normal conditions, the inactive eIF2‐GDP is recycled back into the active form (eIF2‐GTP) by eIF2B‐mediated guanine nucleotide exchange. The phosphorylation of eIF2α increases the affinity of eIF2B to eIF2, sequestering eIF2B, inhibiting the GTP recycling action of eIF2B. This results in the initiation of the downstream elements of the integrated stress response (ISR) and inhibition of cap‐mediated translation initiation resulting in a global shut off of translation (Figure 1b) and SG formation (Figure 1c; Asano et al., 2000; Pavitt & Ron, 2012; Sudhakar et al., 2000). Even a small increase in eIF2 phosphorylation can inhibit global protein translation as the cellular level of eIF2B is 10–20 times lower than that of eIF2 (Montero et al., 2008). To allow a stress‐resolving gene expression program, a subset of mRNAs remain translated following eIF2α phosphorylation, via alternative modes of translation initiation (Figure 1d). These include transcripts containing either an internal ribosome entry site (IRES), possessed by 10% of all cellular mRNAs, or short upstream ORFs (uORFs) such as those encoding the transcription factors, activating transcription factor 4 (ATF4), the CCAAT/enhancer‐binding protein homology protein (CHOP), and growth arrest and DNA‐damaged protein 34 (GADD34). ATF4 is a transcription factor that translocates to the nucleus and associates with one of several dimerization partners to induce the transcription of genes required to restore ER homeostasis such as GADD34 (Figure 1e), which facilitates the dephosphorylation of phospho‐eIF2α during the decline of the stress via the recruitment of protein phosphatase 1 (PP1; Connor et al., 2001; Novoa et al., 2001). GADD34 expression is increased, mediated by both transcription and translation following eIF2α phosphorylation. ATF4 binds and trans‐activates a conserved ATF site in the GADD34 promoter enhancing transcription (Ma & Hendershot, 2003). Additionally, GADD34 mRNA is preferentially recruited to the polysomes mediated by its 5′‐UTR (Lee et al., 2009). This functions as a negative feedback loop by which the expression of GADD34 facilitates the reversal of eIF2α phosphorylation and promotes recovery from translation inhibition (Brush et al., 2003; Novoa et al., 2001; Rojas et al., 2015). Under nonstress conditions, the constitutive repressor of eIF2α (CreP), a constitutively expressed regulatory subunit of PP1 maintains low levels of phosphorylated eIF2α, especially at the ER (Figure 1f; Jousse et al., 2003; Kastan et al., 2020). CreP was shown via proximity labeling to anchor the translation initiation machinery to the ER by binding eIF2α, ensuring localized protein synthesis. This localization is thought to aid translation activity at the ER during stress‐induced global inhibition of translation (Kastan et al., 2020).

#### 2.2.2 Inhibition of eIF4F assembly

Viruses can antagonize the interactions underpinning the eIF4F complex assembly and its interaction with 43S particles by cleavage of eIF4G, separating the eIF4E‐interacting domain from the eIF4A‐ and eIF3‐binding segment, thereby abolishing eIF4F assembly and therefore cap‐dependent translation. PABP is also targeted by viral proteases inhibiting its interaction with the 3′‐polyadenylated tail and association with eIF4G thereby inhibiting translation (SG inhibition by viral proteases will be discussed in Section 5.2; Walsh et al., 2013). Many viruses target eIF4F assembly indirectly by inducing the hypophosphorylation of the eIF4E binding protein 1 (4E‐BP1), which sequesters eIF4E, through the activation of the Akt‐mammalian target of rapamycin (mTOR) signaling axis. The activity of eIF4E is regulated by a family of eIF4E‐binding proteins, referred to as 4EBPs (von der Haar et al., 2004). Under normal conditions, nutrients activate the mechanistic target of rapamycin (mTOR), a kinase of subunit of the mTORC1, which phosphorylates 4EBPs at multiple sites functioning as a sensor of nutrient availability. Although under stress conditions the 4EBPs become hypophosphorylated (McCormick & Khaperskyy, 2017; Zoncu et al., 2011). In this inhibitory state, 4EBPs bind eIF4E preventing its association with eIF4G, resulting in inhibition of eIF4F complex assembly, which is required for cap‐mediated translation inhibition (von der Haar et al., 2004).

Many viruses lack cap structures or polyadenylated tails, therefore use alternate initiation mechanisms and subvert cellular translation initiation by altering the subcellular localization of translation factors to inhibit cap‐dependent translation. This includes the recruitment of eIF3 to viral replication compartments during Sindbis virus (SV) infection, or the nuclear retention of eIF4E and PABP seen during infection with rotavirus and Rift Valley fever virus (RVFV; Blakqori et al., 2009; Burgess et al., 2022; Harb et al., 2008; Sanz et al., 2009; Sukarieh et al., 2010). For a review of viral manipulation of RNA decay, modification and translation see (Burgess et al., 2022).

#### 2.2.3 Translation inhibition by mRNA regulation

Finally, viruses can directly target mRNAs to inhibit translation. Influenza virus (Dias et al., 2009; Guilligay et al., 2008; Plotch et al., 1981; Shih & Krug, 1996) and hantavirus (Garcin et al., 1995; Reguera et al., 2010) encode endonucleases, which cleave host mRNAs downstream of the m^7^GTP cap, hijacking the cap for use on their own viral RNAs. This facilitates viral RNA transcription, destabilizing host mRNAs, and inhibiting cellular translation (Walsh et al., 2013). Similarly, SARS‐CoV‐1 and 2 nonstructural protein 1 (nsp1) possess a two‐pronged strategy to induce host shut‐off and associate with 40S ribosomes, blocking the mRNA entry site and inducing cleavage of host mRNAs (Huang et al., 2011; Kamitani et al., 2006, 2009; Schubert et al., 2020; Simeoni et al., 2021; Thoms et al., 2020). Overall, these strategies culminate in the disassembly of polysomes and actively translating ribosomes from mRNAs, which acts as the first step in mediating SG assembly as outlined in the following sections.

## 3 RNA GRANULES AND PHASE SEPARATION IN VIRAL AND CELLULAR PROCESSES

RNA granules are ubiquitous, microscopically visible, nonmembrane bound condensates formed via self‐assembly of RNA‐binding proteins and RNA by phase separation. These cytoplasmic RNP structures have important roles in posttranscriptional regulation of gene expression by controlling RNA translation and stability (Hyman et al., 2014). Examples include SGs and processing bodies (PBs), each characterized by specific components alluding to their functions. SGs contain small ribosomal subunits, translation initiation factors, and RNA binding proteins (RBPs) such as G3BP1/2 (Kedersha & Anderson, 2002; Tourrière et al., 2003). PBs contain markers including those of mRNA decay (XRN1, DCP1), mRNA surveillance (UPF1, SMG5), and translation regulation (eIF4E, DDX6; Eulalio et al., 2007; Standart & Weil, 2018). SG are induced in response to a cellular stress and disassemble rapidly following resolution of the stress, whereas PBs are present in every cell and part of the mRNA life cycle (Q. Zhang, Sharma, et al., 2019). Despite being constitutively present, PB assembly is a tightly regulated process and both SG and PBs can greatly differ in size and number depending on the conditions. Both SG and PB demonstrate antiviral properties and therefore are targets for viral manipulation (Beckham & Parker, 2008; Poblete‐Duran et al., 2016; Q. Zhang, Sharma, et al., 2019). This review will focus on the viral manipulation of SGs (Viral PB regulation is recently reviewed here (Kanakamani et al., 2021).

### 3.1 Introduction to phase separation processes

RNA granules assemble by LLPS mechanisms, in which protein and protein‐laden RNAs that are dispersed in the cytoplasm and nucleoplasm (soluble phase) coalesce into a concentrated state (condensed phase; C. P. Brangwynne et al., 2009). Phase separation corresponds to the demixing of a homogenous mixture in solution into two separated phases, such as oil droplets separating from water. This allows the formation of sub‐compartments containing complex chemical environments in liquid‐like droplets that can coexist with the surrounding cytoplasm with the constituents of both phases remaining in dynamic equilibrium (Hyman et al., 2014; Yoo et al., 2019). Cells require compartmentalization to organize complex biochemical reactions in the relatively large cytoplasmic space. Phase separated compartments differ from cell membrane bound compartments such as mitochondria (Friedman & Nunnari, 2014) and lysosomes (Luzio et al., 2007) in that they do not have a defined outer wall and the contents can diffuse freely. At low concentration, the constituents of RNA granules remain in a dispersed state, although when enough crosslinks form and the percolation threshold is reached, LLPS occurs. The percolation threshold is defined as the concentration above which individual proteins and protein‐laden RNAs become populated with sufficient crosslinks to form a system‐spanning network (Harmon et al., 2017). Many membrane‐less cellular compartments are known to form via phase separation (Lau et al., 2020). These include nucleoli in the nucleus in which ribosomes are created (Boisvert et al., 2007), centrosomes which nucleate microtubules (Mahen & Venkitaraman, 2012), Cajal bodies in which spliceosomes are made (Gall, 2003), paraspeckles, and nuclear speckles which regulate gene expression (Fox et al., 2018), PBs for RNA storage and processing (Luo et al., 2018) and SGs which form in the cytoplasm in response to different cellular stresses (Buchan & Parker, 2009; Decker & Parker, 2012; Guillén‐Boixet et al., 2020). P‐granules of *C*. *elegans* were the first described liquid–liquid phase‐separated compartment shown to possess liquid‐like properties (Wolf et al., 1983). Upon collision, two granules combine and return to a spherical shape. They can also be formed via dripping off from nucleoli, were shown to constantly exchange material with the cytoplasm (ribosomes are synthesized in the cytoplasm and assembled in the nucleoli) and to internally rearrange, when fully or partially photobleached respectively (C. P. Brangwynne et al., 2009; Hyman et al., 2014). Phase separation rapidly increases component concentration, therefore increasing the rate of collisions and reactions that can take place while simultaneously the segregation of components from the cytoplasm can inhibit reactions from taking place. An example of this, is the depletion of mTORC1 from the cytoplasm into SGs, which can be released to enable signaling upon activation of the dual‐specificity tyrosine phosphorylation regulated kinase 3 (DYRK3) which causes SGs to dissolve (Wippich et al., 2013). Phase separation can involve a single protein solution, two or more proteins, or RNA forming a complex (Alberti et al., 2009). These proteins, like prions, are enriched in IDRs, which are low complexity regions, featuring repetitive amino acid sequences (Alberti et al., 2009; Lau et al., 2020; Oldfield & Dunker, 2014; Wootton & Federhen, 1996). Prions form stable structures, whereas phase separating proteins form much more dynamic structures which can assemble and disassemble rapidly (Chakravarty & Jarosz, 2018). The repetitive amino acid sequences of proteins containing IDRs confer multivalency, which is essential for phase separation as it can make multiple interactions forming a crosslinked network (Banani et al., 2017; Boeynaems et al., 2018). IDRs are enriched in amino acid side chains with biased properties such as being uncharged, charged, or aromatic. Therefore, they can form a variety of interactions within the same protein structure, such as hydrogen bonds, pi–pi interactions [noncovalent interaction commonly between unsaturated (poly)cyclic molecules or side chains with free‐electron clouds (pi‐orbitals)], and pi–cation interactions which are essential for phase separation (Qamar et al., 2018; Sherrill, 2013; Vernon et al., 2018). A prime example is fused in sarcoma (FUS), an RNA binding protein associated with SGs. FUS contains an RGG domain, which is enriched in positively charged arginine and glycine, therefore this binds negatively charged RNA (Oldfield & Dunker, 2014; Qamar et al., 2018). FUS phase separates under low salt concentration and when in solution with RNA as this RNA‐binding domain forms a scaffold to accelerate condensate assembly (Fan & Leung, 2016). Similarly, phase separation of P‐granules is driven by the N‐terminus low‐complexity RGG domain of the DDX3 RNA helicase, LAF‐1 in *C*. *elegans*. This condensate is also influenced by salt concentration highlighting the weak and reversible nature of the interactions involved in liquid droplets that can be dispersed by an increase in electric charge (Elbaum‐Garfinkle et al., 2015). Despite the importance of IDRs in phase separation, RNA‐recognition motifs (RRMs) were found to be the primary modulators for the phase separation of poly‐uridylate binding protein as when the RRMs were removed and the IDR remained, condensate formation was inhibited (Kroschwald et al., 2018).

### 3.2 Phase separation of viral components during infection

Many viral proteins are capable of LLPS and contain IDRs. These intrinsically disordered proteins (IDPs) are different from classical proteins in that they do not fold into a defined stable 3D structure and exist as a highly dynamic and heterogeneous conformational ensemble (Brocca et al., 2020). The lack of a defined structure allows IDPs to bind multiple partners and serve multiple roles, making them ideal for viruses in which the genome size is restrained (Xue et al., 2010, 2014). In contrast to the folding funnel of ordered proteins, IDPs have a flat energy landscape, and contain polar and charged residues, referred to as disorder promoting residues, rather than order‐promoting hydrophobic or bulky residues (Chong & Ham, 2019; Mao et al., 2010; Radivojac et al., 2007). The folding funnel of ordered proteins assumes that the native state (folded state) of a protein corresponds to its free energy minimum, and therefore folding is driven by decreased entropy. LLPS by viral proteins have been shown to promote the formation of viral replication factories and nonreplicative membrane‐less organelles, which interfere with host cell functions (Brocca et al., 2020).

#### 3.2.1 Inclusion bodies

The first account of LLPS of viral protein in vivo corresponds to the description of Negri bodies in neurons infected with rabies virus (RABV). These Negri bodies were found to be inclusion bodies (IBs) (Nikolic et al., 2017). Similarly, vesicular stomatitis virus (VSV, also a rhabdovirus; Lahaye et al., 2009), Ebola virus (EBOV, a filovirus; Hoenen et al., 2012), and respiratory syncytial virus (RSV, a pneumovirus; Rincheval et al., 2017) all form bona fide IBs as they each possess all the viral ribonucleoparticles and RNA that are synthesized inside these particles. In addition, some viral proteins have been found to be able to induce LLPS of IB‐like condensates alone or by co‐expression. For example, the co‐expression of the P and N proteins of paramyxoviruses leads to the formation of spherical condensates which recapitulate the liquid properties of IBs (Nevers et al., 2020). Similarly, the N protein of EBOV is sufficient to form IBs whereas VSV requires the co‐expression of the L protein (Miyake et al., 2020). In each case, the protein regions required for IB formation are intrinsically disordered (Brocca et al., 2020).

#### 3.2.2 Phase separation during virion packaging and egress

The use of LLPS by viruses is not limited to replication. The second IDR of influenza A virus (IAV) nucleoprotein has been shown to interact with viral RNA and phosphatidylinositol (4,5) bisphosphate (PI(4,5)P2) at the plasma membrane during the packaging of the eight viral genome segments, which must be compacted together in single copies during replication (Alenquer et al., 2019; Kakisaka et al., 2016). Several recent studies have also highlighted the ability of the coronavirus N proteins, including that of SARS‐CoV‐2 to phase separate with the addition of RNA or the M protein (Cai et al., 2021; Carlson, Asfaha, Ghent, Howard, Hartooni, & Morgan, 2020; Carlson, Asfaha, Ghent, Howard, Hartooni, Safari, et al., 2020; Cascarina & Ross, 2020; Cubuk et al., 2020; Dang et al., 2021; Iserman, Roden, Boerneke, Sealfon, McLaughlin, Jungreis, Park, et al., 2020; Iserman, Roden, Boerneke, Sealfon, McLaughlin, Jungreis, Fritch, et al., 2020; Jack et al., 2020; Lu et al., 2021; Luo et al., 2021; T. M. Perdikari et al., 2020a, 2020b; Roden et al., 2021; Savastano et al., 2020; Wang et al., 2021; Wu et al., 2021; Zhao et al., 2021). In addition, N has been shown to interact with the 5′ UTR and the region corresponding to the putative packaging signal. It is suggested to aid in viral packaging by the condensing of the viral genome and interacting with the membrane‐associated M protein to bud new virions (Lu et al., 2021). All these examples highlight how viruses take advantage of phase separation processes to favor their replication.

## 4 STRESS GRANULES

**FIGURE 2:**
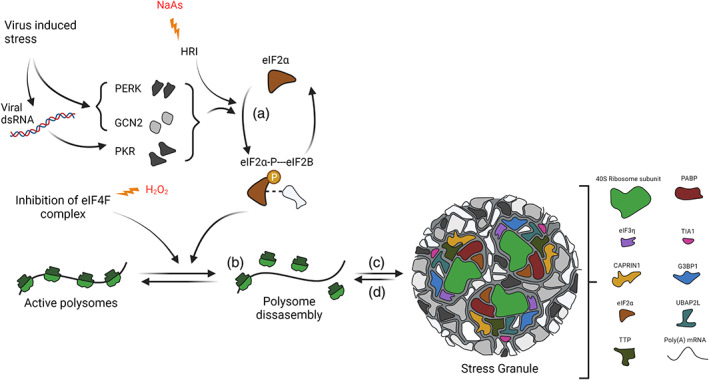
Schematic of eIF2‐dependent stress granule induction. (a) Phosphorylation of eIF2α (p‐eIF2α), mediated by an eIF2α kinase. (b) Translation initiation is inhibited (stalled) following the recruitment of p‐eIF2α to the translation initiation complex. (c) Formation of a stress granule following the aggregation of stalled initiation complexes in combination with SG regulatory proteins including G3BP1, TIA‐1, and CAPRIN‐1. (d) SG disassembly occurs rapidly upon resolution of stress and translation can resume. SG contain translationally silent mRNA, 40S ribosomal subunits, eukaryotic initiation factors (e.g., eIF4G, eIF4A, and eIF3), and RNA binding proteins [e.g., G3BP1, cell cycle‐associated protein 1 (CAPRIN1), human antigen R (HuR), ubiquitin‐specific peptidase 10 (USP10), UBAP2L, and T‐cell intracellular antigen 1 (TIA1)]. Figure made using Biorender.

SGs are dynamic cytoplasmic condensates ranging from 0.1 to 2 μm, formed to resolve various environmental stresses and serve as sites of mRNA storage and triage (Anderson & Kedersha, 2008; White & Lloyd, 2012). SGs were first described in plant cells (Nover et al., 1983) but have been most extensively studied in mammalian cells [Plant SG reviewed by Maruri‐López et al. (2021)]. SG assembly can be triggered by oxidative stress, heat shock, osmotic shock, or viral infection (Aulas et al., 2017; Khong et al., 2017; Namkoong et al., 2018; Panas et al., 2016; Protter & Parker, 2016) and usually follows the phosphorylation of eIF2α discussed in Section 2.2.1 (Figure 2a). This then leads to translation inhibition and the subsequent polysome run‐off and release of free mRNAs (Figure 2b,c; Anderson & Kedersha, 2002; Kedersha et al., 2002). This increase in free RNA is essential for SG assembly and in fact when polysome disassembly is inhibited by cycloheximide, SG formation is blocked (Kedersha et al., 2000). SGs contain translationally silent mRNA, 40S ribosomal subunits, eukaryotic initiation factors (e.g., eIF4G, eIF4A, and eIF3), and RNA binding proteins [e.g., G3BP1, cell cycle‐associated protein 1 (CAPRIN1), human antigen R (HuR), ubiquitin‐specific peptidase 10 (USP10), UBAP2L, and T‐cell intracellular antigen 1 (TIA1); Buchan & Parker, 2009; Yang et al., 2020; Figure 2). SGs disassemble upon stress resolution and reinitiation of translation (Figure 2d). SG formation can also be achieved via eIF2α‐independent pathways such as cleavage of the eIF4G scaffold protein or inhibition of eIF4E helicase, thereby also disrupting translation initiation (Lloyd, 2012). SGs have also been found to interact and exchange components with other membrane‐less organelles in cytoplasm, such as P‐bodies, or in the nucleus as shown with paraspeckles [crosstalk between RNP granule sis reviewed by An et al. (2021)].

### 4.1 SG assembly and composition

#### 4.1.1 SG assembly

**FIGURE 3:**
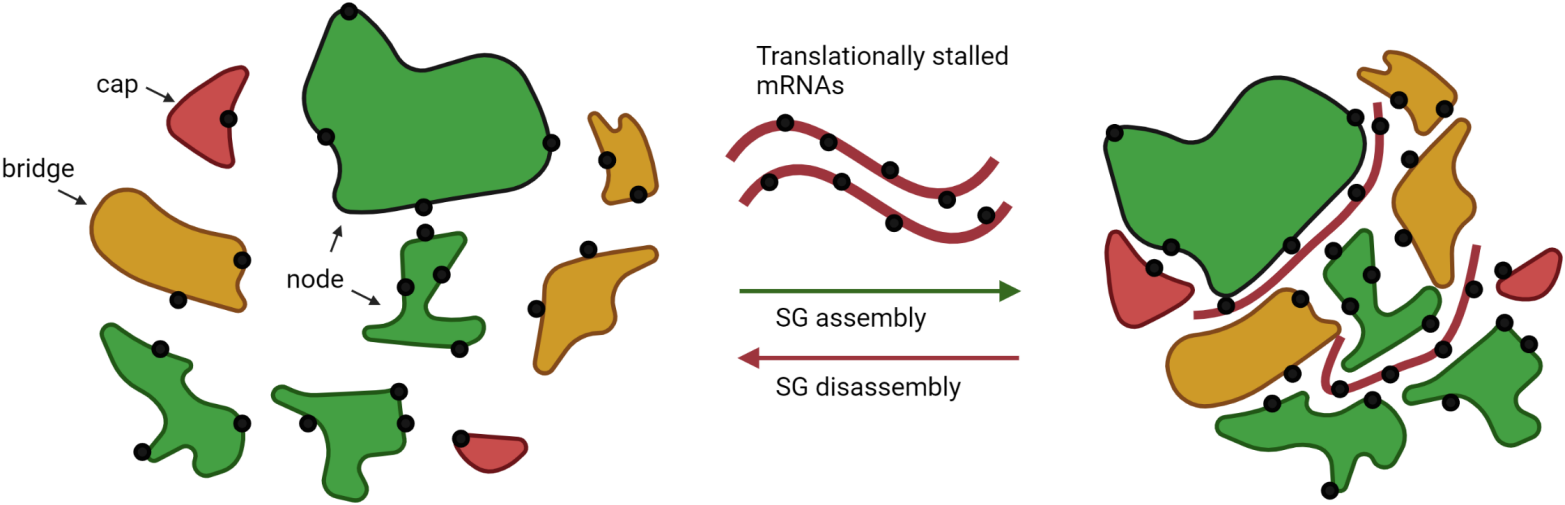
Schematic detailing stress granule assembly via the network‐based cellular condensation model. Nodes (blue) are defined as macromolecules that have three or more interaction (v ≥ 3) domains (eg. G3BP1), which are essential to drive LLPS due to increased multivalency allowing network formation. Bridges are proteins that can make two interactions (*v* = 2) and caps are defined as a protein that can make one interaction (*v* = 1), therefore acting as a dead end and limiting network growth (Hofmann et al., 2021)

SGs assemble via LLPS events in which dispersed RNAs or RNAs bound to proteins, aggregate into a condensed phase (C. P. Brangwynne et al., 2009). SG assembly is initiated by an increase in the concentration of cytoplasmic free mRNA that accompanies inhibition of translation after release from polysomes. This increase in mRNA concentration is then sensed by G3BP1 which acts as a molecular switch to activate SG assembly by RNA dependent LLPS (Freibaum et al., 2021; Gal et al., 2019; Guillén‐Boixet et al., 2020; Matheny et al., 2021; Panas et al., 2016; Protter & Parker, 2016; Sanders et al., 2020; Yang et al., 2020). G3BP1 is present in an auto‐inhibited compact conformation in unstressed cells. This compact state is held by electrostatic interactions between the positively charged arginine‐rich region and the acidic intrinsically disordered tracts (Guillén‐Boixet et al., 2020). Released mRNA from the polysomes competitively inhibits this compact state, inducing a conformational change that allows the clustering and crosslinking of G3BP1 and RNA into networked RNA–protein condensates. Further recruitment of SG proteins contributes to reaching a threshold saturation concentration, termed the percolation threshold, initiating LLPS, and seeding SG assembly (Guillén‐Boixet et al., 2020). The percolation threshold of SG assembly is defined by the network of protein–protein, protein–RNA, and RNA–RNA interactions that drive LLPS (Guillén‐Boixet et al., 2020; Sanders et al., 2020; Yang et al., 2020). The constituents of this network each contribute toward reaching the required percolation threshold for LLPS although different components contribute more than others (Figure 3; Yang et al., 2020). There are ~36 proteins that contribute to the percolation threshold in SG. Node proteins are multivalent RNA‐binding proteins (*v* ≥ 3) that can cross‐link and accelerate SG assembly. Bridges can form two connections (*v* = 2) and caps form one (*v* = 1), therefore these slow or prevent SG growth, respectively (Figure 3; Yang et al., 2020).

The most central nodes of the SG network are G3BP1 and G3BP2 which initiate SG assembly by undergoing LLPS with RNA (Guillén‐Boixet et al., 2020; Sanders et al., 2020; Yang et al., 2020). In agreement with this, knockout of both G3BP1 and G3BP2 inhibit SG assembly in response to NaAs treatment (Kedersha et al., 2016). In contrast to previous belief, SG were still able to form in cells with double knockout of paralogous isoforms of proteins previously deemed essential for SG assembly including UBAP2, UBAP2L, TIA1, and TIAR (Yang et al., 2020). Therefore, G3BP1/2 remains the only SG resident protein deemed essential for NaAs induced SGs. Despite this SG can still be formed in cells devoid of G3BP1/2 when induced by heat shock and osmotic stress, which indicates that the driving stimulator dictates the factors driving SG assembly (Yang et al., 2020).

#### 4.1.2 SG reconstitution

It was shown recently that SG‐like condensates could be reconstituted in U2OS lysates with the addition of purified G3BP1 and that these lysates were most similar to NaAs induced granules (Freibaum et al., 2021), whereas induction of LLPS by TIA1, FUS, and TAR DNA‐binding protein‐43 (TDP‐43) did not reconstitute SG (P. Zhang, Fan, et al., 2019). It has been shown that RNA‐dependent LLPS required both the dimerization domain (nuclear transport factor 2‐like, NTF2L) and the RNA binding domain (RBD) of G3BP1, as when these domains are knocked out RNA induced LLPS was not achieved (Yang et al., 2020). The addition of the eight amino acid FGDF motif of nsp3 of CHIKV, which interacts with the NTF2L domain of G3BP1, is sufficient to inhibit LLPS by G3BP1, highlighting the importance of increased valency of G3BP1 in undergoing LLPS (Fros et al., 2012; Panas et al., 2014). Reconstituted G3BP‐RNA condensates were shown to have relatively low numbers of G3BP in relation to RNA and that not all binding sites on the RNA were saturated. It is thought that these condensates act as RBP recruiting centers. In support of this, CAPRIN‐1 or TIA‐1 were shown to reduce the saturation concentration of G3BP1‐RNA condensates by a concept called polyphasic linkage. In this case, preferential binding of a ligand (CAPRIN‐1) to scaffold molecules (G3BP) in the condensate leads to a downshift in saturation concentrations (Guillén‐Boixet et al., 2020; Wyman & Gill, 1980; Yang et al., 2020). It is thought that this phenomenon may extend to other SG proteins such as UBAP2L, cold shock domain‐containing E1 (CSDE1), and PRRC2C1 (Guillén‐Boixet et al., 2020). It was shown that features of RNA that influence LLPS included long length, being single‐stranded, and RNA–RNA interactions, as when total RNA was pretreated with the RNA helicase, DDX19A, and ATP, in vitro LLPS of G3BP1 was strongly inhibited (Yang et al., 2020). The interaction of G3BP1 with different SG proteins can positively or negatively regulate assembly. For example, UBAP2L, CAPRIN‐1, and TIA increase assembly but USP10 limits assembly (Kedersha et al., 2016; Markmiller et al., 2018; Yang et al., 2020).

#### 4.1.3 SG composition and tools for the study

The composition of SG can vary depending on the stage the mRNA translation cycle is stalled. These can be classified into three subtypes [type I–type III; Reviewed in Advani and Ivanov (2020), Ivanov et al. (2019), and C. L. Riggs et al. (2020)]. Type I canonical SGs form in response to phosphorylation of eIF2α, requires G3BP1 and 48S preinitiation complexes, but lack eIF2 and eIF5. Type I SGs assemble in response to oxidative stress, ER stress, and viral infection (Kedersha et al., 2002). Inhibition of translation via an eIF2α‐independent route results in type‐II SG. These SGs are formed post joining of the eIF2‐loaded ternary complex to the 48S preinitiation complex and therefore these SG contain eIF2 and eIF5. These SGs can be formed using eIF4F inhibitors via inactivation of eIF4A. Finally, type III SG lack eIF3 and assemble in response to glucose starvation, sodium selenite, nitric oxide, and UV (Advani & Ivanov, 2020; P. Anderson et al., 2015; Aulas et al., 2018; Fujimura et al., 2012). Yet to be fully compositionally characterized is another type of noncanonical SG assembled in response to osmotic stress. These SGs can form independently of G3BP1 and eIF2α‐phosphorylation and their assembly is thought to be aided by molecular crowding, defined as the condensation of protein/RNA (Bevilacqua et al., 2010; Bounedjah et al., 2012; Ivanov et al., 2019; Youn et al., 2018).

Fluorescence recovery after photobleaching (FRAP) experiments have demonstrated the rapid exchange of many SG components, some with half‐lives of less than 30 s, despite this, other components were more immobile (Buchan & Parker, 2009). It was demonstrated by super‐resolution microscopy that SGs formed during NaAs treatment are nonuniform and contain stable substructures called cores which are surrounded by a less concentrated dynamic shell (Jain et al., 2016; Niewidok et al., 2018; Wheeler et al., 2016). It was recently shown that UBAP2L forms distinct cores separate from G3BP1 and authors suggest a model in which UBAP2L nucleates SGs upstream of G3BP1 (Cirillo et al., 2020). Due to the increased stability of SG cores, it is possible to purify them from U2OS cells expressing GFP tagged G3BP1 (Khong et al., 2018; Wheeler et al., 2017). Purification and RNA‐Seq analysis of cores (Jain et al., 2016), as well as proximity labeling (Markmiller et al., 2018; Youn et al., 2018), revealed a diverse SG proteome including many RBPs which form a dense protein–protein interaction network (Jain et al., 2016). In addition to protein–protein interactions, RNA–RNA interactions have been shown to contribute toward the RNA composition of SGs [reviewed in Van Treeck and Parker (2018)]. Protein‐free RNA assemblies formed in vitro from total yeast RNA showed a high degree of overlap with the transcriptome of yeast SGs, indicating an important role of RNA–RNA interactions in recruitment of RNA or RNPs to condensates (Van Treeck et al., 2018). Transcriptomic analysis of immunopurified SGs revealed a disproportionate enrichment of some mRNAs (AHNAK and NORAD), and the relative exclusion of others (glyceraldehyde 3‐phosphate dehydrogenase, GAPDH; Khong et al., 2017). It was also shown that the partitioning of RNPs into SG is increased by RNA length and inhibited by association with ribosomes as long, AU‐rich, and poorly translated transcripts have been demonstrated to preferentially enrich in SGs (Khong et al., 2017; Matheny et al., 2019; Namkoong et al., 2018).

### 4.2 Current understanding of SG disassembly routes

SG rapidly disassemble upon resolution of the causative stress [SG disassembly reviewed in Hofmann et al. (2021)]. Drugs that stabilize polysomes such as cycloheximide and emetine result in SG disassembly (Anderson & Kedersha, 2006; Kedersha et al., 2000), as SG require the constant exchange of mRNA with the cytosol (Kedersha & Anderson, 2009; Parker & Sheth, 2007). Cycloheximide and emetine are translation elongation inhibitors and therefore trap mRNPs into the polysomes. Cycloheximide binds the large ribosomal subunit, 60S, in the E site, stalling elongation by preventing exit of the deacetylated E‐site tRNA. Emetine binds the small ribosomal subunit, 40S, in the E site preventing translocation of the tRNA–mRNA complex (Hobson et al., 2020). In contrast to the assembly of SG, less is known about SG disassembly. Recovery from artificial SG assembly such as NaAs, hydrogen peroxide (H_2_O_2_) treatment, osmotic stress, and heat shock takes from 60 to 120 min (Hofmann et al., 2021), whereas cold shock recovery can take only a few minutes (Anderson & Kedersha, 2009). G3BP1 has two major binding partners CAPRIN‐1 and USP10, the binding sites of which overlap, therefore, the binding of one competitively inhibits the other (Kedersha et al., 2016). CAPRIN‐1 binding G3BP1 induces SG assembly and USP10 binding results in SG disassembly (Kedersha et al., 2016; Sanders et al., 2020).

SG are thought to be regulated by posttranslational modifications such as arginine methylation, by which UBAP2L is regulated (Huang et al., 2020), phosphorylation of G3BP1, or growth factor receptor‐bound protein 7 (Grb7; Guillén‐Boixet et al., 2020; Tsai et al., 2008) and PARylation. PARylation is the posttranscriptional modification in which polymers have the covalent addition of poly ADP‐ribose (Duan et al., 2019; Leung et al., 2011, 2012). It has been shown that phosphorylation of G3BP1 at sites S149 and S232 reduces the ability of G3BP1 to phase separate. This was proposed to strengthen the auto‐inhibitory closed conformation of G3BP1 which reduces the valency and RNA binding of G3BP1 (Guillén‐Boixet et al., 2020). It has previously been shown that CK2 phosphorylates G3BP1 at S149 to downregulate SG formation (Reineke et al., 2017). In addition, phosphorylation of Grb7 was shown to promote disassembly of SG induced by heat shock (Tsai et al., 2008).

SG are also thought to be modulated by chaperones or disaggregates such as valosin‐containing protein (VCP; Kroschwald et al., 2015; Seguin et al., 2014; Wang et al., 2019) and heat shock protein 90 (HSP90; Mediani et al., 2021). VCP is an AAA‐ATPase that interacts with ubiquitinated proteins via cofactors allowing their removal from protein complexes or membranes for degradation and recycling by the proteasome (Meyer et al., 2012; Meyer & Weihl, 2014). Unc‐51 like autophagy activating kinase 1 (ULK1) and ULK2 have been shown to regulate SG disassembly through phosphorylation and activation of VCP (Wang et al., 2019) and HSP90 has been shown to bind and stabilize the DYRK3, which promotes SG disassembly upon stress resolution when active (Mediani et al., 2021; Wippich et al., 2013). In addition, it has been shown that RNA helicase eIF4A reduces the condensation of RNA in vitro via an ATP‐dependent RNA binding process limiting SG formation (D. Tauber et al., 2020).

### 4.3 SGs and antiviral signaling

Cells have evolved highly specialized stressing mechanisms that detect viral products and actively suppress the majority of global translation to counteract virus hijacking of host translation, interconnecting the ISR and innate immune sensing. Growing evidence suggests that the ISR, especially SGs, contribute to antiviral defense (reviewed by Eiermann et al. (2020)]. Upon viral infection of a cell, nucleic acids such as DNA, RNA, and replication intermediates are released into the cytoplasm. These are termed pathogen‐associated molecular patterns (PAMPs) and are identified as nonself by immune sensors. This activates an antiviral state through the production of IFNs and pro‐inflammatory cytokines (Goubau et al., 2013; Jensen & Thomsen, 2012; Ma et al., 2018). Upon infection, dsRNA accumulates in the cytosol, and viral proteins in the ER trigger stress sensors PKR and PERK, respectively (Donnelly et al., 2013). Activation of these kinases initiates the ISR by phosphorylation of eIF2α leading to translation inhibition, polysome disassembly, and the induction of selected genes, including ATF4, which promote cellular recovery (Pakos‐Zebrucka et al., 2016). Viral nucleic acids may also differ in their 5′‐cap structure which is detected by PRRs. Although, many viruses encode their own 2′O‐methyltransferases to avoid detection, some cleave and commandeer host cap structures or use RNA secondary structural motifs to antagonize IFIT1 binding (Hyde & Diamond, 2015). SG are frequently observed upon viral infection. Once considered to be sites of mRNA triage and storage (Anderson & Kedersha, 2008; Buchan & Parker, 2009). SG are now also considered as signaling platforms given their enrichment in RBPs and signal transducing factors, bringing together sensors and effectors (Kedersha et al., 2013; Mahboubi & Stochaj, 2017; McCormick & Khaperskyy, 2017; Onomoto et al., 2014).

#### 4.3.1 Innate immune sensing and antiviral signaling

Cells possess a number of innate immune sensors; these include the RIG‐I‐like receptors (RLRs) family including RIG‐I and MDA5. RLRs signal via the mitochondrial‐associated adaptor protein MAVS. The endosomal TLRs signal via TRIF and MyD88, and cytosolic DNA sensors (CDSs) such as Z‐DNA binding protein 1 (ZBP‐1), cyclic GMP‐AMP synthase (cGAS), DDX41, and interferon‐gamma inducible protein 1 (IFI16) signal via the ER adaptor molecule stimulator of interferon response CGAMP interactor (STING) (Abe & Barber, 2014; Dobbs et al., 2015; Kawai et al., 2005; Liu et al., 2015; Seth et al., 2005). All of the above signaling pathways converge on the activation of the transcription factors, IRF 3 and 7 by the TANK‐binding kinase 1 (TBK1) and NFκB by the IκB kinase (IKK) complex (Jensen & Thomsen, 2012; Ma et al., 2018; Majzoub et al., 2019; Tan et al., 2018). Type I IFN genes are induced following the nuclear translocation of the phosphorylated forms of IRF 3 and 7. IFN secretion leads to IFN‐receptor activation and Janus kinase/signal transducers and activators of transcription (JAK/STAT) signaling (Ivashkiv & Donlin, 2014) which leads to the transcriptional activation hundreds of interferon stimulating genes with antiviral activity (Schoggins et al., 2011), including PKR, all of which contribute to the formation of an antiviral state.

Aside from translation inhibition, PKR has been linked to other immune sensor signaling pathways including cGAS, RIG‐I (Yoo et al., 2014), and MDA5 (Liu et al., 2019; Pham et al., 2016). The cytosolic DNA sensor, cGAS, and G3BP1 were shown to form a complex necessary for dsDNA sensing and IFN production (Liu et al., 2019). It was also demonstrated that PKR‐induced condensates were required for efficient cGAS DNA sensing (Hu et al., 2019). Additionally, PKR promoted RIG‐I activation in response to IAV and Newcastle disease virus (NDV) infection (Yoo et al., 2014) and associated with MDA5 in response to vaccinia virus (VACV) infection to stimulate IFN‐β production via the MAVS/IRF3/7 signaling cascade (Pham et al., 2016).

#### 4.3.2 The impact of SGs in antiviral signaling, and as effectors of antiviral functions

The antiviral nature of SGs can be demonstrated by knocking out viral mechanisms to antagonize SG formation. For example, when NS1 of IAV was mutated, its PKR antagonism was lost, this resulted in the accumulation of viral RNA in avSGs along with RIG‐I, MDA5, OAS, RNaseL, and PKR. Redistribution of innate sensors such as RIG‐I is also observed for encephalomyocarditis virus (EMCV), adenovirus, and SINV, and in addition, when SG were knocked out by depletion of SG components, IFN production was greatly reduced, and viral replication was increased. These results prompted Onomoto et al. to propose the term avSGs as they serve as a platform where innate immune sensors can be activated by viral RNA to initiate the IFN response and an antiviral state (Onomoto et al., 2012). Similar findings are seen for other viruses such as EMCV, which induces SG at early timepoints. These SGs contain MDA5 and dsRNA, which are later inhibited by the cleavage of G3BP1, resulting in reduced IFNβ and cytokine response (Ng et al., 2013). Recently, SG formed during Infectious bronchitis virus (IBV) infection were demonstrated to be avSGs as the IRF3‐IFN response was attenuated and viral replication was increased upon infection of SG‐defective cells. In addition, RRLs such as PKR, MDA5, TLR3, and signaling intermediates, MAVS, TNF receptor‐associated factor 3 (TRAF3), TRAF6, TBK1, and IKKε, were present in SG formed during infection in 20% of infected cells, suggesting that avSGs function as a scaffold for viral RNA recognition by RLRs in wild‐type infection by IBV (Gao et al., 2021). RIG‐I and (Onomoto et al., 2012) MDA5 (Langereis et al., 2013) also localize to SG formed due to NaAs and heat stress indicating that MDA5/RIG‐1 can also assemble with endogenous RNAs indicating that they localize to SG even without viral RNA. However, some of the evidence associating antiviral signaling and SG remains debatable, in particular, whether the association of SG‐resident proteins with SGs themselves is truly required for their antiviral properties as recently discussed in Eiermann et al. (2020) and Mateju and Chao (2021). There is a growing body of data implicating the ISR and SG components in antiviral defense. Although it is important to separate whether this is a SG‐dependent or independent function, which is difficult to control. For example, CAPRIN, G3BP1, and G3BP2 have been demonstrated to contribute to the translation of IFN stimulated genes independently of SG assembly (Bidet et al., 2017). Similarly, G3BP1 has been shown to be involved in innate immune responses and cytokine expression (Reineke & Lloyd, 2015), to stimulate IFN production and cytokine expression by binding viral dsRNA and enhancing RIG‐I (Kim et al., 2019; W. Yang, Ru, et al., 2019) and by activating cGAS independently of SG assembly (Liu et al., 2019). Therefore, G3BP1 clearly has a role in the detection of viral genomes. Despite this, a large degree of evidence does suggest SG assembly is a contributing factor to the antiviral response. For example, the ability of G3BP1 to inhibit enterovirus replication was linked to SG formation using G3BP1 deletion mutants (Reineke & Lloyd, 2015). Furthermore, the interaction of G3BP1 with PKR and its subsequent SG localization has been linked with increased PKR activity and decreased viral replication (Reineke et al., 2015; Reineke & Lloyd, 2015). Additionally PKR activity has been shown to be disrupted following pharmacological disruption of SG assembly or depletion of SG components (Burgess & Mohr, 2018; Reineke et al., 2015). Therefore, the majority of literature does support the model of SG as antiviral platform although the specific virus and conditions may have a contributing role (Eiermann et al., 2020; Mateju & Chao, 2021).

## 5 VIRAL STRATEGIES TO ANTAGONIZE STRESS GRANULES

As previously discussed, viruses have evolved a plethora of strategies to counteract the innate immune response and SG assembly, to efficiently replicate their genome. These strategies include antagonism of the PKR/PERK‐eIF2α axis, cleavage of essential SG components or sequestration, and repurposing of SG components (Q. Zhang, Sharma, et al., 2019).

### 5.1 Evasion of PKR as a SG counteracting measure

Most viruses that antagonize the ISR have evolved strategies to reverse or inhibit the phosphorylation of eIF2α and the resulting assembly of SGs. These strategies allow the evasion of IFN induction by dsRNA sensing and PKR activation of eIF2α and OAS, which induces RNaseL, resulting in mRNA and ribosomal RNA (rRNA) degradation (Schneider & Mohr, 2003; Walsh et al., 2013). Antagonists of the ISR are classified from I to IV based on the antagonistic mechanism they use (examples are collated in Table 1). Class I antagonists avert eIF2α kinase activation by blocking or sequestering the stressor. Class II antagonists prevent eIF2α phosphorylation by binding and inhibiting the activated sensor. Class III antagonists induce the dephosphorylation of eIF2α by activating the PP1 phosphate. Class IV antagonists recently proposed by Rabouw et al., inhibit the p‐eIF2:eIF2B interaction allowing the continued formation of the GTP–Met–tRNAi ternary complex and unrestricted translation (Rabouw et al., 2020).

**TABLE 1:** Evasion of PERK/PKR—Class I–IV

Genome	Virus family	Virus	Viral protein	Strategy	References
dsDNA	*Poxviridae*	Vaccinia virus (VACV)	K3L	Prevents eIF2‐p by binding and inhibiting the function of the activated sensor (Class II)	(Dar & Sicheri, 2002; Kawagishi‐Kobayashi et al., 1997)
	*Herpesviridae*	Herpes simplex virus (HSV)	Gamma 34.5 protein	Induce eIF2a dephosphorylation by PP1 (Class III)	(Li et al., 2011)
	*Asfarviridae*	African swine fever virus (ASFV)	V16 and F18 residues in DP71L protein	Induce eIF2a dephosphorylation by PP1 (Class III)	(Zhang et al., 2010)
(+)ssRNA	*Picornaviridae*	Aichivirus–Picornavirus	AiVL	Competitive inhibition of p‐eIF2–eIF2B interaction (Class IV)	(Rabouw et al., 2020)
	*Coronaviridae*	Infectious bronchitis virus (IBV)	Nsp2?	Upregulation of GADD34 expression dephosphorylate eIF2α‐P	(Wang et al., 2009)
		Transmissible gastroenteritis virus (TGEV)	p7	Induce eIF2a dephosphorylation by PP1 (Class III)	(Cruz et al., 2011)
		Beluga whale CoV (Bw‐CoV)	ORF10	Competitive inhibition of p‐eIF2–eIF2B interaction (Class IV)	(Mihindukulasuriya et al., 2008; Rabouw et al., 2020)
	*Flaviviridae*	Herpes simplex virus (HCV)		Oscillation of SG by upregulation of GADD34	(A. Ruggieri et al., 2012)
		Zika virus (ZIKV)		Upregulation of GADD34 expression dephosphorylate eIF2α‐P	(Amorim et al., 2017)
(−)ssRNA	*Paramyxoviridae*	Parainfluenza virus type 3 (HPIV)		IB shield viral RNAs (Class I)	(Hu et al., 2018)
	*Hantaviridae*	Andes virus	N	Nucleocapsid protein interrupts PKR dimerization (Class I)	(Wang & Mir, 2015)
(+)ssRNA‐RT	*Retroviridae*	Human immunodeficiency virus 1 (HIV‐1)	Staufen 1 Gag	Inhibits PKR and eIF2a phosphorylation (Class II) Prevent SG formation by interaction with Staufen 1	(Abrahamyan et al., 2010; Rao et al., 2018; Rao et al., 2019)

**TABLE 2:** Viral proteases that inhibit stress granules

Genome	*Virus family*	Virus	Viral protein	Strategies	References
(+)ssRNA	*Picornaviridae*	EV71	2A or L,3C	Cleaves eIF4GI and G3BP1 later in infection (3C). Interferes with the eIF4GI–G3BP1 interaction	(Yang et al., 2018; X. Yang, Hu, et al., 2019; Zhang et al., 2018) (Chang et al., 2017)
		Seneca valley virus	3C	Disruption of eIF4GI–G3BP1 interaction	(Wen et al., 2020)
		PV	3C	Cleaves G3BP1, eIF4GI, and eIF4GII	(White et al., 2007) (Black et al., 1989)
		FMDV	L	Cleaves G3BP1 and G3BP2	(Visser, Medina, et al., 2019)
		EMCV	3C	Cleaves G3BP1	(Visser, Langereis, et al., 2019)
	*Caliciviridae*	FCV	NS6	Cleavage of G3BP1	(Humoud et al., 2016)
(+)ssRNA‐RT	*Retroviridae*	HIV‐1/HIV‐2	HIV‐1Pro	Cleaves eIF4G, GCN2	(Alvarez et al., 2003; Balvay et al., 2007; del Pino et al., 2012; Ventoso et al., 2001)
		SIV		Cleaves eIF4GI	(Balvay et al., 2007)
		MoMLV	MoMLV protease	Cleaves eIF4GI and eIF4GII	(Balvay et al., 2007)
		MMTV	MMTV protease	Cleaves eIF4GI, eIF4GII, and PABP	(Balvay et al., 2007)

#### 5.1.1 Class I: Avert eIF2α kinase activation

Viruses can apply a variety of strategies to avert eIF2α kinase activation. Some viruses encode dsRNA binding proteins, competing with, and inhibiting PKR activation. Viruses encoding dsRNA binding proteins include VACV E3L protein (Chang et al., 1992), human cytomegalovirus (HMCV) TRS‐1 and IRS‐1 proteins (Child et al., 2004; Hakki & Geballe, 2005), reovirus σ3 (Beattie et al., 1995), HSV‐1 Us11 (Mulvey et al., 1999), and MERS‐CoV 4a (Comar et al., 2019; Nakagawa et al., 2018; Rabouw et al., 2016).

The C‐terminus of VACV E3L protein is a dsRNA‐binding domain that when mutated or deleted is sufficient to attenuate the virus, which is used as a vaccine candidate (Denzler et al., 2011; Vijaysri et al., 2008). HCMV TRS‐1 and reovirus σ3 were separately inserted into VACV lacking the E3L gene to show their dsRNA binding effects (Beattie et al., 1995; Child et al., 2004; González‐López et al., 2003). The accessory protein 4a of MERS‐CoV inhibits the dsRNA‐mediated activation of PKR by binding dsRNA. This action was found to be specific to PKR, as 4a was not able to inhibit the activation of eIF2α via other eIF2α kinases including, NaAs, heat stress, or pateamine A. Interestingly without 4a expression, MERS‐CoV was still able to inhibit SG formation, therefore MERS‐CoV possesses at least one other strategy for SG suppression (Rabouw et al., 2016). Another strategy for the aversion of eIF2α phosphorylation and SG formation is the shielding of the stressor. IAV possesses a dsRNA binding protein, NS1, which shields dsRNA from detection by RLRs and PKR while a virus lacking NS1 failed to inhibit PKR and IRF3 activation (Lu et al., 1995; Talon et al., 2000).

CoV nsp15 has been demonstrated to reduce free dsRNA in the cytoplasm avoiding cytoplasmic sensors (Gao et al., 2021). Similarly, CoVs (Doyle et al., 2018; Knoops et al., 2008; Maier et al., 2013, 2016; Ulasli et al., 2010) and many other viruses (Mackenzie, 2005; Netherton et al., 2007; Novoa et al., 2005) induce membrane rearrangements which can shield the viral replication by‐products from detection and activation of PKR and the RNAseL pathway. For example, newly synthesized RNA is concealed from detection by IBs formed during parainfluenza type 3 virus (HPIV3) infection. These IBs are induced by the interaction between the nucleoprotein (N) and phosphoprotein (P) of HPIV3 and were demonstrated to suppress SG formation. Furthermore, mutation of N preventing association with P prevented IB formation and SG inhibition (Hu et al., 2018).

As an alternative to binding the substrate (dsRNA), the adenoviral RNA‐I (VAI) binds a single monomer of PKR preventing its dimerization and activation by viral dsRNA, therefore keeping it in its inactive state (Launer‐Felty et al., 2015). VAI is a short noncoding RNA with an apical stem loop, a highly structured central domain, and a terminal stem (Launer‐Felty et al., 2015).

The L protein the picornavirus Theiler's murine encephalomyocarditis virus (TMEV) inhibits SG formation by an unknown mechanism, and without the cleavage of G3BP1 unlike other picornaviruses (Borghese & Michiels, 2011; Langereis et al., 2013). While eIF2α phosphorylation is inhibited during infection, the L protein impairs SG assembly following arsenite challenge without blocking eIF2α phosphorylation. Moreover, in cells infected with a mutant‐L virus PKR colocalized with viral dsRNA but not in wild‐type virus infection, suggesting that the L protein indirectly inhibits the activation of PKR by blocking the PKR–dsRNA interaction (Borghese et al., 2019).

#### 5.1.2 Class II: Prevent eIF2α phosphorylation by binding an activated sensor

Following eIF2α kinases activation, viruses can prevent the downstream phosphorylation of eIF2α. Poxviruses such as VACV, myxoma virus, and smallpox virus express PKR inhibitors. K3L of VACV and M156R of myxoma virus closely resemble the N‐terminus OB‐fold domain of eIF2α and therefore act as pseudosubstrate inhibitors, a decoy, of PKR and GCN2 (Qian et al., 1996; Ramelot et al., 2002). While K3L shares 28% amino acid sequence identity with the N terminus of eIF2α, the C‐terminus of K3L is homologous to residues 79–83 in eIF2α, adjacent to the phosphorylation site at Ser‐51, and the truncation or points mutation within these sites abrogates the inhibition of PKR (Kawagishi‐Kobayashi et al., 1997; Seo et al., 2008).

#### 5.1.3 Class III: Induce dephosphorylation of eIF2α by recruiting the cellular phosphatase PP1

GADD34 interacts with PP1 through an RVxF domain to dephosphorylate eIF2α (Novoa et al., 2001). ICP34.5 of HSV1 mediates dephosphorylation of eIF2α by binding PP1 via its RVxF motif (Bryant et al., 2008; He et al., 1997, 1998). Similarly, accessory protein 7 (p7) of TGEV also interacts with PP1 via its RVxF motif to dephosphorylate eIF2α. Mutant virus lacking p7 induced a host translational shut off, enhanced apoptosis and nuclease activity, further suggesting an increase in dsRNA‐activated host responses (Cruz et al., 2011). Zika virus (ZIKV) inhibits SG formation by inducing the dephosphorylation of eIF2α by upregulating of GADD34 expression (Amorim et al., 2017). Similarly, eIF2α phosphorylation is suppressed during IBV infection which has also been linked with increased GADD34 expression (Wang et al., 2009). Furthermore, the autophosphorylation of PKR is also suppressed during infection by IBV nsp2 (Wang et al., 2009). Finally, oscillating SGs have been observed during HCV infection, in which repeating cycles of SG assembly and disassembly follow corresponding cycles of translation inhibition and re‐entry, respectively. HCV presents as a chronic infection therefore these oscillations are thought to prevent long‐lasting translation repression allowing the persistence of the cell during infection without cell death (A. Ruggieri et al., 2012). The disassembly of SG has been linked to upregulation of GADD34 expression, which rapidly increases upon stress exposure, although protein amounts are tightly controlled due to the short half‐life (≤1 h) of the protein. GADD34 is rapidly degraded by the proteasome likely owing to its nature as a IDP, therefore having little 3D structure (Brush & Shenolikar, 2008; Choy et al., 2015; Zhou et al., 2011). SG oscillation cycles are maintained by the balancing act of PKR activation and eIF2α phosphorylation to shut off translation and assembly SGs, which in turn induces GADD34 expression and eIF2α dephosphorylation to restore translation and disassemble SGs. Oscillating SGs have also been reported during infection with NDV and Sendai virus (SeV) and is suggested to be a hallmark of RNA virus infection (A. Ruggieri et al., 2012).

#### 5.1.4 Class IV: Competitive inhibition of p‐eIF2–eIF2B interaction

At the core of the ISR is the sequestration of eIF2B by p‐eIF2α, which prevents eIF2 recycling and inhibits global protein synthesis. Recently, two viruses were discovered to have independently acquired the ability to specifically block the p‐eIF2α:eIF2B interaction. The open reading frame 10 (ORF10) encoded accessory protein (AcP10) of the recently identified beluga whale coronavirus, SW1 (Mihindukulasuriya et al., 2008), and AiVL of the human gastroenteric picornavirus, Aichivirus (Rabouw et al., 2020) are able to block this eIF2B sequestration allowing unhindered formation of the eIF2–GTP–Met–tRNAi ternary complex and translation, despite the high levels of eIF2α phosphorylation (Rabouw et al., 2020). AcP10 inhibits the assembly of NaAs‐induced SGs, whereas NS1 of IAV, a dsRNA binding protein, and class I antagonist, was not able. AcP10 is also unable prevent eIF2α phosphorylation upon NaAs treatment, a class II property, and does not dephosphorylate eIF2α, a class III property. Additionally, AcP10 was not able to inhibit SG induced by pateamine A which induces SG in an ISR‐independent manner (Rabouw et al., 2020). As part of the ISR, ATF4 is expressed from uORF2 upon translation inhibition. When AcP10 was co‐expressed with a GFP reporter controlled by the ATF4 regulatory element under stress conditions, ATF4 expression was inhibited, whereas ATF4 expression was rescued upon expression of an AcP10 mutant. This indicated that AcP10 allows continued ternary complex formation despite high eIF2α phosphorylation (Rabouw et al., 2020). AcP10 was found to interact with both eIF2 and eIF2B, and associated with eIF2Bε in the high salt conditions that disrupt the eIF2:eIF2B complex. Therefore, AcP10 interacts with eIF2B to inhibit eIF2B sequestration and ISR activation, while allowing eIF2B and eIF2 interactions to continue ensuring unhindered translation initiation. Similar findings were also described for AiVL of Aichivirus highlighting a conserved mechanism in unrelated viruses (Rabouw et al., 2020).

### 5.2 Cleavage of SG or UPR components by viral proteases

Viruses encode proteases important for the processing of viral polyproteins as summarised on Table 2, yet having evolved alongside host proteins, many have gained cellular antagonistic functions. These include the cleavage of SG components such as G3BP1/2 (G3BP), eIF4G, PABP1, or cellular sensors such as PKR and GCN2 [reviewed by Q. Zhang, Sharma, et al. (2019)].

#### 5.2.1 Viral proteolysis of SG components

**FIGURE 4:**
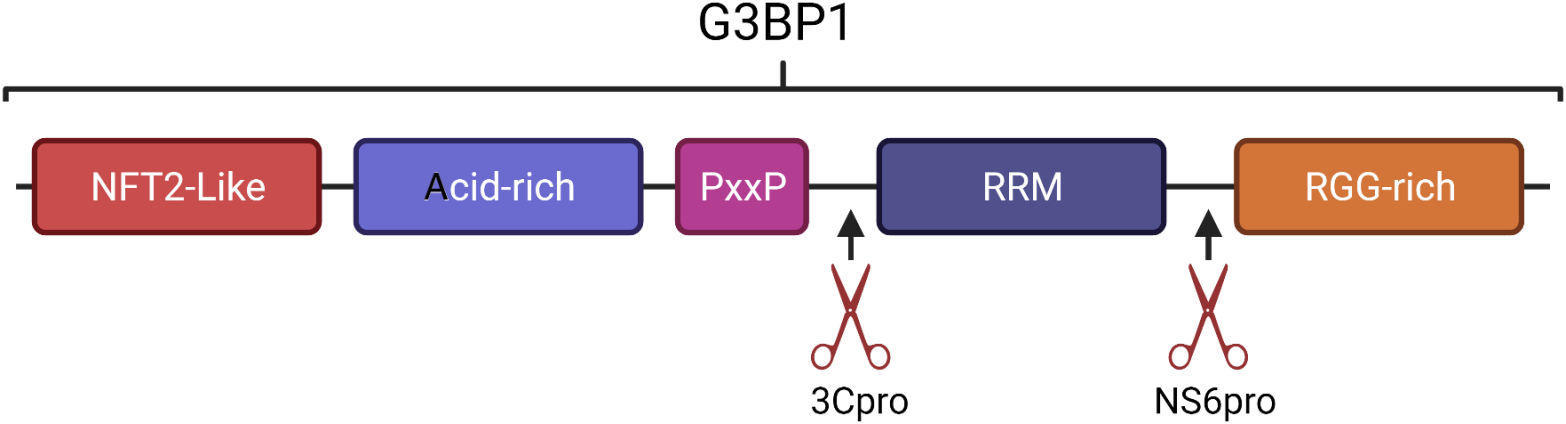
Schematic of G3BP1 and cleavage sites by FCV and PV. G3BP1 contains several functional domains, an N‐terminus NFT2‐like domain, central PxxP motifs, and two RNA binding domains (RBD; RRM motif and RGG motif) at the C‐terminus. Both feline calicivirus (FCV) and poliovirus (PV) cleave G3BP1 inhibiting SG assembly. FCV 3C‐like protease NS6^pro^ cleaves G3BP1 at site E405/V406 between the two RBDs whereas poliovirus 3C cleaves at site Q305/G326 removing both RBDs. Adapted from White et al. (2007) cleavage sites and motif sizes are approximate (Humoud et al., 2016). Created with BioRender.com

SG assembly can be antagonized via cleavage of SG nucleating proteins. Many viral proteases target the essential SG nucleating protein G3BP1. These include poliovirus (PV) 3C (White et al., 2007), EMCV (Ng et al., 2013), Enterovirus 71 (EV71) 3C (Zhang et al., 2018), NS6^Pro^ of feline calicivirus (FCV; Humoud et al., 2016) and foot and mouth disease virus (FMDV) leader protease (Visser, Medina, et al., 2019). PV and EMCV both induce SGs early in infection and inhibit them later via G3BP1 cleavage by 3C (Ng et al., 2013; White et al., 2007). Early in PV infection, SGs contain G3BP1, eIF4G, and PABP (White et al., 2007), which are all later cleaved by 3C or 2A, resulting in remaining atypical SGs (aSGs) that contain TIA‐1 (Piotrowska et al., 2010). The enterovirus EV71 also inhibits SG via 3C‐mediated G3BP1 cleavage later in infection. Interestingly, the initial SG induction was found to be due to the 2A protease. These 2A‐induced SG are aSGs given that 2A blocks typical SG (tSG) formation through the cleavage of eIF4G disabling the eIF4GI‐G3BP1 interaction. These aSG are devoid of G3BP1, and several eIFs, could not be dissolved using cycloheximide, and were independent of eIF2α phosphorylation. Interestingly cellular mRNAs are sequestered into aSGs, unlike viral mRNA which is released and available for viral replication (W. Yang, Ru, et al., 2019; Yang et al., 2018; Zhu et al., 2016). FCV also cleaves G3BP1, with its 3C‐like protease NS6^Pro^, impairing SG formation despite the presence of phosphorylated eIF2α (Humoud et al., 2016). Interestingly, FCV NS6^Pro^ cleaves G3BP1 at the E405/V406 junction, which differs from the PV 3C Q325/G326 target sites (Figure 4). In consequence, the 3C‐like protease cleaves G3BP1 between its C‐terminus RRM And RGG RNA binding domains, 3C proteases cleaves off both (Humoud et al., 2016).

Similar to the complexity of SG inhibition by different picornaviruses, different retroviruses also inhibit SG and host translation using multiple strategies. HIV‐1, HIV‐2, SIV (complex retroviruses), Moloney murine leukemia virus (MoMLV), and mouse mammary tumor virus (MMTV; simple retrovirus) all cleave eIF4GI and SIV, MoMLV, and MMTV also cleave eIF4GII. Similarly, to picornaviruses and caliciviruses, HIV‐1, HIV‐2, and MMTV also cleave PABP abrogating cellular cap‐mediated translation while allowing IRES‐mediated viral translation (Alvarez et al., 2006; Balvay et al., 2007; Butsch & Boris‐Lawrie, 2000; Ventoso et al., 2001).

#### 5.2.2 Viral proteolysis of eIF2α kinases

SG assembly can also be inhibited via the degradation of eIF2α kinases. Viral proteases, including PV and EV71 3C, can cleave the main virus‐induced eIF2α kinase PKR (Chang et al., 2017) (Black et al., 1989). Although typically associated with amino acid starvation and activation of the general amino acid control pathway, GCN2 can be also activated in response to viral replication by tRNA and viral RNA (Berlanga et al., 2006; Brocard et al., 2021; Dever et al., 1992; Zhang et al., 2002). GCN2 was also shown to bind several retroviral integrase and may function as a inhibitor of foreign DNA integration (Jaspart et al., 2017). Viral inhibition as a result of GCN2 activation, is seen for many viruses including HIV‐1/2 (del Pino et al., 2012), SINV (Berlanga et al., 2006), Dengue virus (DENV; Afroz et al., 2020), murine norovirus (MNV; Brocard et al., 2021), and VSV (Krishnamoorthy et al., 2008). Cells were shown to be more susceptible to VSV, SINV (Berlanga et al., 2006), and DENV (Afroz et al., 2020) infection when GCN2 was knocked out, suggesting an antiviral role of GCN2 against these viruses. It was also shown that GCN2 is activated by HIV‐1 viral RNA and this is overcome by the proteolytic cleavage of GCN2 by HIV‐1 protease (HIV‐1^pro^; Balvay et al., 2007; Clerzius et al., 2011; del Pino et al., 2012; Jaspart et al., 2017).

### 5.3 Sequestration or repurposing of SGs components to counteract SGs assembly

#### 5.3.1 Sequestration of SG proteins by viral components

**FIGURE 5:**
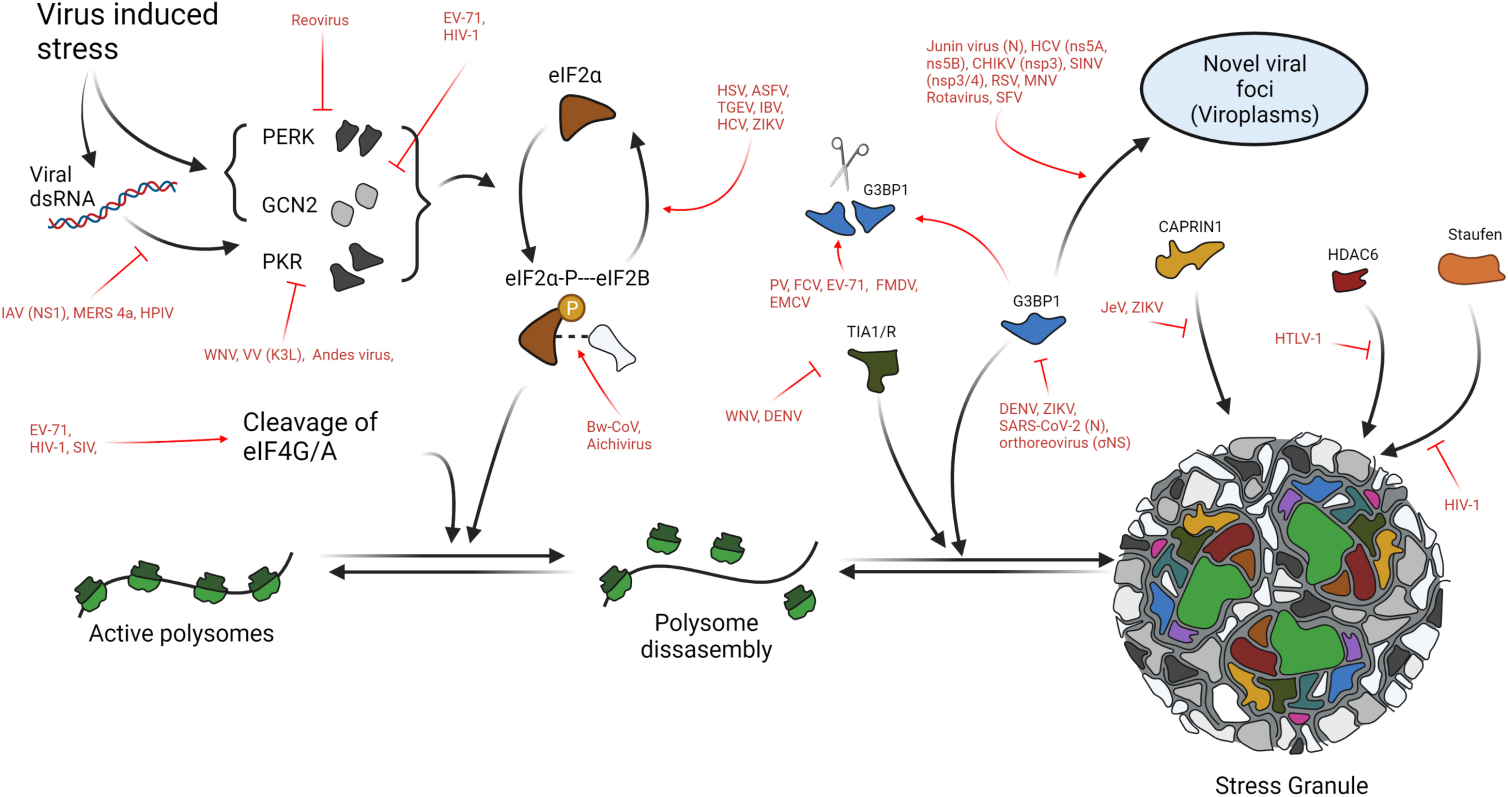
Schematic of viral stress granule antagonism. Viral strategies to inhibit translation and SGs as described in Tables 1, 2, 3 are summarized in the schematic above. Created with BioRender.com

In addition to sequestration of dsRNA and eIF2α kinases, other SG components such as G3BP1 can be sequestered preventing SG formation. Some viruses subvert SG components and relocate them to sites of viral replication (SG inhibition by sequestration is summarized in Table 3, and Viral antagonism of SG is summarized in Figure 5).

**TABLE 3:** Stress granule inhibition by sequestration of components

Genome	Virus family	Virus	Strategy	References
dsRNA	*Reoviridae*	Rhesus monkey rotavirus and human rotavirus	Changes G3BP1 and ZBP1 localization	(Dhillon & Durga Rao, 2018)
		Mammalian orthoreovirus	σNS protein associates with G3BP1 to disrupt SGs	(Choudhury et al., 2017)
(+)ssRNA	*Flaviviridae*	JeV	Interacts with CAPRIN‐1 to block SG formation–Core protein	(Katoh et al., 2013)
		ZIKV	Alters the cellular localization of HuR, and capsid proteins form complexes with G3BP1 and CAPRIN‐1	(Bonenfant et al., 2019) (Hou et al., 2017)
		West Nile and Dengue	Interact with TIA and sequester from SG—ends of viral minus‐strand RNA	(Emara & Brinton, 2007)
	*Caliciviridae*	MNV	Recruits G3BP1 to the viral replication complex	(Fritzlar et al., 2019)
	*Togaviridae*	SFV	Sequester G3BP1 by forming complex—nsp3	(Panas et al., 2012)
		CHIKV and many other alphaviruses	Sequester G3BP1 into cytoplasmic foci (aSG)—nsp3	(Nowee et al., 2021)
	*Coronaviridae*	SARS‐CoV‐2	Interacts with and sequesters G3BP1 inhibiting SG assembly—N protein	(Luo et al., 2021; Nabeel‐Shah et al., 2020; Zheng et al., 2021)
(−)ssRNA	*Arenaviridae*	Junin virus	N protein colocalizes with G3BP and replication–transcription complexes	(Linero et al., 2011)
(+)ssRNA‐RT	*Retroviridae*	HTLV‐1	Interaction of viral Tax protein with histone deacetylase 6 (HDAC6)	(Legros et al., 2011)
		HIV‐1	Gag colocalizes with staufen‐1	(Abrahamyan et al., 2010; Rao et al., 2019)

SG‐like particles or IBs are formed by many viruses including CHIKV and SFV, these have been shown to be non‐bona fide SG as they do not possess eIF3 (Fros et al., 2012; Panas et al., 2012). In fact, nsp3 of CHIKV, which makes up part of the replication complex effectively sequesters G3BP1, inhibiting SG assembly (Fros et al., 2012). This interaction has been shown to be essential for viral replication as well as inhibitory to the SG‐mediated antiviral response (Scholte et al., 2015). Many alphaviruses have been shown to interact with G3BP1 or its mosquito homolog, Rasputin [RIN; reviewed in Nowee et al. (2021)]. Nsp3 can also sequester fragile X mental retardation protein (FMRP), which is also a SG protein (Mazroui et al., 2002) and has been implicated in the assembly of SG (Didiot et al., 2009). G3BP1 contains a NTF2L domain, involved in SG formation (Tourrière et al., 2003). Several alphaviruses have mimicked G3BP1‐binding domains such as FGxF motifs in their hypervariable domain. This allows inhibition of SG formation via G3BP1 sequestration as this domain is originally found in CAPRIN‐1 and is important for SG nucleation (Cristea et al., 2006; Fros et al., 2012; Gorchakov et al., 2008; Kim et al., 2016; Panas et al., 2012, 2014, 2015).

Like FCV, the calicivirus MNV also prevents SG formation (Humoud et al., 2016). However, MNV infection does not induce G3BP1 cleavage and instead results in the rewiring of the G3BP1 protein interactome by relocating G3BP1 to replication complexes, simultaneously stimulating translation of the viral RNA and replication, while inhibiting SG formation. As a result, suppression of G3BP1 negatively impacted viral replication (Brocard et al., 2021; Fritzlar et al., 2019; Hosmillo et al., 2019). Furthermore, norovirus VPg protein has been demonstrated to interact with G3BP1 to facilitate VPg‐dependent viral translation by association with the component of eIF4F complex (Hosmillo et al., 2019). Flaviviruses such as DENV, west Nile virus (WNV; Emara & Brinton, 2007), Japanese encephalitis virus (JeV; Katoh et al., 2013), and ZIKV (Amorim et al., 2017; Hou et al., 2017) have all been shown to inhibit SGs. The JeV core protein sequestrates CAPRIN‐1 (Katoh et al., 2013), while DENV and WNV relocate the SG components TIAR and TIA1 to viral replication complexes (Emara & Brinton, 2007). Interestingly, WNV and other flavivirus infection induce reactive oxygen species (ROS) although infected cells are resistant to NaAs SG induction. WNV infection was shown to increase the expression of cellular antioxidants, and this was sufficient to inhibit NaAs SG induction (Basu et al., 2017). In addition to inducing the dephosphorylation of eIF2α phosphorylation by upregulating GADD34 expression (Amorim et al., 2017), the ZIKV capsid, NS3, NS2B‐NS3, and NS4A, also inhibit SGs by forming stable complexes with G3BP1 and CAPRIN‐1 (Hou et al., 2017). Interestingly G3BP1 was demonstrated to have a positive effect on ZIKV replication as depletion of G3BP1 resulted in decreased gene expression and virion production. In contrast depletion of another SG component, HuR, increased ZIKV replication, therefore individual SG components can have antiviral effects aside from their role in SG (Bonenfant et al., 2019). HTLV‐1 targets yet another SG component to inhibit SG. The Tax protein inhibits the formation of SG by interacting with histone deacetylase 6 (HDA6) (Legros et al., 2011).

#### 5.3.2 Competition between cellular and viral phase‐separated compartments to disable SG assembly

Upon cell entry, viruses are confronted with the hostile environment of host innate immunity (as discussed in Section 4.3.1). SG are formed upon sensing of viral dsRNA by PKR and are considered antiviral as they serve as signaling platforms for innate immunity, condensing RLRs, effectors and viral RNAs can together with PKR induce the IFN response. In agreement with this, viral replication is usually either increased or unchanged when SG are inhibited (Q. Zhang, Sharma, et al., 2019). Despite this, some viruses induce SG‐like IBs or viroplasms which sequester SG components, therefore, inhibiting SGs. These IBs do not represent bona fide SGs as they are often missing essential components. IBs are observed during infection by CHIKV (Nowee et al., 2021), EBOV (Nevers et al., 2020), and Semliki forest virus (SFV; Panas et al., 2012; These are summarized and discussed in Section 3.2.1). SARS‐CoV‐2 N protein interacts and recruits cellular protein G3BP1 to N condensates but does not recruit other SG associated proteins such as Ubiquitin associated protein 2‐like (UBAP2L), DEAD‐box helicase 1 (DDX1), and eIF3η. This likely represents the interference of cellular innate immunity and SG assembly and indeed this interaction has been shown to inhibit SG formation (Cai et al., 2021; Lu et al., 2021; Luo et al., 2021; Wang et al., 2021). Interestingly, host mRNA are also sequestered into N condensates and this activity is regulated by the phosphorylation of the N protein, with increased host mRNA localization to unphosphorylated N protein (Nabeel‐Shah et al., 2020). Therefore SARS‐CoV‐2 also regulates the translation of host mRNAs.

## 6 CONCLUSION

Here, we reviewed the interplay between viruses and SGs and various counteracting strategies have been discussed. Many recent studies have highlighted the potential antiviral nature of SGs, yet despite this several viruses have been described that appear to replicate alongside them. This coexistence often appears to be neither beneficial nor severely deleterious to viral replication, for example, during RABV infection in which Negri bodies neighbor and exchange mRNA with SG (Nikolic et al., 2016). Conversely, other viruses such IBV and yellow fever virus (YFV) feature seemingly avSGs during infection that only occur in a small proportion of infected cells (Beauclair et al., 2020; Brownsword et al., 2020; Gao et al., 2021). SGs formed during YFV were recently shown to be uncoupled from the acute cytokine response and RIG‐1 and PKR activity was unaffected by SG disassembly (Beauclair et al., 2020). These data, together with a current understanding that SGs assembled in response to different stressors are heterogeneous, raise further questions. How truly compositionally and functionally different are SGs that form early during infection by enteroviruses such as PV, EV71, or CVB3, from those formed during infection with IBV and YFV, or those assembling in an oscillatory pattern during HCV infection remains unknown (A. Ruggieri et al., 2012). Future studies should aim at applying the robust ‐omics pipelines recently established to uncover the identity of SG resident proteins and RNAs, whether through their affinity purification (Jain et al., 2016), proximity‐ligation approaches (Reineke & Lloyd, 2015), or spatial proteomics (Marmor‐Kollet et al., 2020). This will shed unprecedented light into fundamental differences between what could be classified as antiviral or proviral SGs. In addition, while most studies described have relied on the use of cell lines expressing fluorescent reporter SG markers, more studies are needed within the context of either infected tissues or in relevant primary cells. This may further inform our understanding of SG biogenesis, for example, recent data suggest that ROS may have a role in the production of SG, as hydrogen peroxide has been shown to be present in SG induced by NaAs (Hu et al., 2021). In support of this, WNV was shown to inhibit SG via upregulation of the antioxidant, glutathione (Basu et al., 2017). Conversely, SG may instead have roles in the sequestration and suppression of ROS to inhibit apoptosis and recover from nonlethal stress (Takahashi et al., 2013). These examples and those previously detailed describe a complex staging ground that can be manipulated or avoided by the virus in a number of ways producing perhaps a multitude of compositionally and functionally different granules that may warrant further classification than currently prescribed. Furthermore, the ability of viruses to condense their IDR‐rich proteins by LLPS into membrane‐less organelles has been recently demonstrated by SARS‐CoV‐2 N and MeV in which both colocalize with RNA and contribute to an efficient replication system. Many viruses, SARS‐CoV‐2 included, also associate with G3BP1 to disassemble SG and in doing so it has become an integral part of their replication cycle. Interestingly a polyphenol from green tea, gallocatechin gallate (GCG) was recently described to inhibit the interaction between the nucleoprotein and RNA of SARS‐CoV‐2, inhibiting LLPS, and viral replication (Hong et al., 2021). Similarly, a condensate hardening drug cyclopamine and its chemical analog, A3E, were shown to alter the liquid‐like properties of RSV IBs resulting in their disorganization and disassembly inhibiting viral replication in vivo (Risso‐Ballester et al., 2021). It is important that in future studies, virus–host interactions between other resident SG proteins are represented as the current literature is lacking to this end. Insight into these interactions is essential to further elucidate the role of these biocondensates and inform targeting strategies for therapeutics. Recent findings have introduced novel granules including RNAse‐L bodies (RLB; Burke et al., 2020) and paracrine granules, the latter induced in cells adjacent to FCV infected cells (Iadevaia et al., 2022). It may therefore be valuable to reevaluate earlier discoveries to determine if these granules have been misidentified and do in fact represent these alternative SG‐like foci. Finally, the notion that a cell can only tolerate a certain degree of phase separation is one that has been touched on in the literature, and emerging studies make clear that viruses have evolved mechanisms of replication and packaging that also depend on IDR rich RBPs and LLPS and indeed those that are integral to SG assembly. As these RBPs are a finite resource, we see this tug of war between the virus and the antiviral response. This emerging dependence of LLPS by viruses may provide us with novel strategies to target conserved processes of viral replication as seen for SARS‐CoV‐2 and RSV.

## AUTHOR CONTRIBUTIONS

**Matthew J. Brownsword:** Conceptualization (equal); formal analysis (lead); investigation (lead); writing – original draft (lead); writing – review and editing (lead). **Nicolas Locker:** Conceptualization (equal); formal analysis (supporting); funding acquisition (lead); project administration (lead); supervision (lead); writing – original draft (supporting); writing – review and editing (supporting).

## FUNDING INFORMATION

Work in N.L.'s laboratory is supported by Biotechnology and Biological Sciences Research Council research grants [grant numbers BB/S006931/1, BB/P068018/1].

## CONFLICT OF INTEREST

The authors declare no competing interests.

## RELATED WIREs ARTICLE

Regulation of stress granules and P‐bodies during RNA virus infection

## Data Availability

Data sharing is not applicable to this article as no new data were created or analyzed in this study.
